# Efficacy and Molecular Mechanisms of Differentiated Response to the Aurora and Angiogenic Kinase Inhibitor ENMD-2076 in Preclinical Models of p53-Mutated Triple-Negative Breast Cancer

**DOI:** 10.3389/fonc.2017.00094

**Published:** 2017-05-15

**Authors:** Anastasia A. Ionkina, John J. Tentler, Jihye Kim, Anna Capasso, Todd M. Pitts, Karen A. Ryall, Rebekah R. Howison, Peter Kabos, Carol A. Sartorius, Aik Choon Tan, S. Gail Eckhardt, Jennifer R. Diamond

**Affiliations:** ^1^Department of Medicine, Division of Medical Oncology, University of Colorado Cancer Center, University of Colorado Anschutz Medical Campus, Aurora, CO, USA; ^2^Department of Pathology, University of Colorado Cancer Center, University of Colorado Anschutz Medical Campus, Aurora, CO, USA

**Keywords:** triple negative breast cancer, ENMD-2076, targeted therapy, aurora kinase A, senescence, resistance mechanisms

## Abstract

**Purpose:**

Triple-negative breast cancer (TNBC) is a subtype associated with poor prognosis and for which there are limited therapeutic options. The purpose of this study was to evaluate the efficacy of ENMD-2076 in p53-mutated TNBC patient-derived xenograft (PDX) models and describe patterns of terminal cell fate in models demonstrating sensitivity, intrinsic resistance, and acquired resistance to ENMD-2076.

**Experimental design:**

p53-mutated, TNBC PDX models were treated with ENMD-2076 and evaluated for mechanisms of sensitivity or resistance to treatment. Correlative tissue testing was performed on tumor tissue to assess for markers of proliferation, apoptosis, senescence, and pathways of resistance after treatment and at the time of acquired resistance.

**Results:**

Sensitivity to ENMD-2076 200 mg/kg daily was associated with induction of apoptosis while models exhibiting intrinsic or acquired resistance to treatment presented with a senescent phenotype. Response to ENMD-2076 was accompanied by an increase in p53 and p73 levels, even within the background of mutant p53. Treatment with ENMD-2076 resulted in a decrease in pAurA and an increase in pHH3. We observed a TNBC subtype switch from the luminal androgen receptor to the basal-like subtype at acquired resistance.

**Conclusion:**

ENMD-2076 has antitumor activity in preclinical models of p53-mutated TNBC. Increased levels of p53 and p73 correlated with sensitivity whereas senescence was associated with resistance to ENMD-2076. The novel finding of a TNBC subtype switch at time of acquired resistance may provide mechanistic insights into the biologic effects of selective pressure of anticancer treatments on TNBC. ENMD-2076 is currently being evaluated in a Phase 2 clinical trial in patients with metastatic, previously treated TNBC where these biologic correlates can be further explored.

## Introduction

Triple-negative breast cancer (TNBC) is an aggressive breast cancer subtype characterized by a lack of estrogen and progesterone receptor expression and HER2 amplification (1–3). Despite a relatively high initial response rate to chemotherapy, patients with TNBC face a higher risk of cancer recurrence and subsequent cancer-related death (4, 5). The treatment of metastatic TNBC remains sequential lines of chemotherapy; however, most tumors develop acquired resistance to treatment quite rapidly (6). The development of targeted anticancer therapies for TNBC remains an unmet clinical need to decrease TNBC-related morbidity and mortality (7).

Aurora kinase A (AurA) is a serine/threonine kinase integral to mitotic cell division (8, 9). Overexpression of AurA in mammary epithelium has been linked to genetic instability, promoting tumor formation, progression, and metastasis (10–12). Aurora kinase inhibitors (AKIs) are active against preclinical TNBC models where exposure leads to a G_2_/M cell cycle arrest followed by aneuploidy and mitotic catastrophe (13–15).

ENMD-2076 is an orally bioavailable, multi-targeted Aurora and angiogenic kinase inhibitor with greater specificity for Aur A [50% inhibitory concentration (IC_50_) 14 Nm] as compared to Aurora B kinase (IC_50_ 350 nM) (16). Additional targets of ENMD-2076 include vascular endothelial growth factor receptor, fibroblast growth factor receptor, and platelet-derived growth factor receptor α (14). ENMD-2076 is active against p53-mutated TNBC cancer cell line models, with more robust apoptotic activity in cell lines with increased p53 expression (14). The purpose of this study was to evaluate the efficacy of ENMD-2076 in p53-mutated TNBC patient-derived xenograft (PDX) models and describe patterns of terminal cell fate in models demonstrating sensitivity, intrinsic resistance, and acquired resistance to ENMD-2076.

## Materials and Methods

### Drugs

The tartrate salt of ENMD-2076 was provided by CASI, Inc. (Rockville, MD, USA) and prepared as a 40 mg/mL stock solution in water for oral gavage administration *in vivo*.

### *In Vivo* TNBC Patient-Derived Xenograft (PDX) Studies

Five- to six-week-old female athymic nude (nu/nu) mice (Envigo, formally Harlan Sprague Dawley) were allowed to acclimate for 1 week prior to handling and housed in groups of five. Animals were provided sterilized food and water *ad libitum* and maintained a 12-h light/dark cycle. PDX models were generated as previously described (14, 15, 17). In brief, tumor tissue was obtained from patients with TNBC treated at the University of Colorado Cancer Center (UCCC) following informed written consent for a Colorado Multiple Institutional Review Board-approved protocol. Following expansion in athymic nude mice, tumor tissue was minced into 5 mm^3^ sections and one tumor was implanted subcutaneously per mouse using a 12-gauge trocar, as previously described (15). Mice were weighed and tumor measurements were collected twice weekly using digital calipers and recorded with the Study Director software package (Studylog Systems). Tumor volume was calculated using the following formula: volume = (length × width^2^) × 0.52. When tumors reached a mean volume of 150 mm^3^, mice were randomized to vehicle and ENMD-2076 groups with at least 10 tumors/group. ENMD-2076 (200 mg/kg) or vehicle control was administered *via* oral gavage with continuous once daily dosing. At the end of treatment, mice were euthanized with isoflurane overdose and tumor samples were collected for gross anatomic, immunohistochemistry (IHC), and immunofluorescence (IF) analysis. Tumor growth inhibition (TGI) was calculated from the average tumor volume of the treated (V_t_) and vehicle control (V_vc_) groups, with the equation: TGI = 1 − (V_t_/V_vc_). All xenograft studies were performed in accordance with the NIH guidelines for the care and use of laboratory animals in a facility accredited by the American Association for Accreditation of Laboratory Animal Care with approval by the University of Colorado Institutional Animal Care and Use Committee prior to initiation of experiments.

### Immunofluorescence

PDX tumor samples were freshly collected from vehicle and ENMD-2076-treated animals at days 4, 30, and at the time of acquired resistance to ENMD-2076 treatment (sensitive models only). Samples were placed into individual cryomolds (Sakura Finetek, Torrane, CA, USA) and embedded in Tissue-Tek^®^ optimum cutting temperature (OCT) Compound (Sakura Finetek, Torrane, CA, USA). Cryomold blocks were frozen in liquid nitrogen and individually cut into 5 µM thick slides by the UCCC Pathology Core Facility. Slides were fixed in a 1:1 ratio solution of methanol and acetone at −20°C for 10 min. Air-dried slides were blocked with 1% BSA in 1× PBS for 1 h at 37°C and subsequently incubated with the appropriate primary antibody [phospho-histone H3 (pHH3), phospho-Aur A (pAurA), p53 (Cell Signaling Technology, Beverly, MA, USA, catalog numbers 3377S, 2914, and 2527S, respectively), BAX, p73, BCL2 (Abcam, Cambridge, MA, USA, catalog numbers 10813, 137797, 16120, respectively), or p16 (Santa Cruz Biotechnology, Santa Cruz, CA, USA, catalog number 468)]. Following three washes in 1× PBS, slides were incubated with secondary antibodies [AlexaFluor 555 (Invitrogen, Carlsbad, CA, USA, catalog number A31572 donkey, A211429 goat) or AlexaFluor 488 (Life Technologies, Carlsbad, CA, USA, catalog number A11055 donkey anti-goat, A21202 donkey anti-mouse, A11015 donkey anti-sheep)] for 1 h at 37°C and then washed three times in 1× PBS. Counterstaining with 300 nM DAPI in 1× PBS for 5 min at room temperature (RT) was performed and subsequently washed twice with PBS for 5 min. Slides were mounted with Fluoromount-G mounting media (SouthernBiotech, Birmingham, AL, USA), cover slipped and sealed with clear nail polish. Slides were imaged using the Olympus FV-1000 confocal microscope at 60× magnification. Images from three different representative fields of view were used to calculate the mean pixel intensity above background. A threshold for background was calculated using the Otsu method (18), defined as the optimum threshold in an intensity histogram that minimizes variance between background and foreground pixels. Image analysis was implemented using Cell Profiler (19).

### Senescence-Associated β-Galactosidase (SA-β-Gal) Assay

Slides obtained from OCT blocks were created as above. Slides were fixed and stained for SA-β-gal activity at a pH of 6.0, using the Senescence β-gal Staining Kit (Cell Signaling Technology, Danvers, MA, USA). Images were acquired using a Zeiss microscope at 40× magnification. Hematoxylin and eosin (H&E) staining was performed on corresponding slides by the UCCC Pathology Core Facility.

### Immunohistochemistry

Formalin-fixed, paraffin-embedded tumor samples were stained using Ki-67 Cline SP6 (Thermo Scientific, catalog number 9106S-1601A), cleaved caspase-3 (Cell Signaling, catalog number 9661), CD34 (Cell Signaling, catalog number GR201207-3 AB81289), androgen receptor (AR, Cell Marque, catalog number 200R-14), or H&E by the UCCC Histology Core Facility. Images were acquired using a Zeiss microscope at 10× magnification. At least three samples from at least two animals were assessed at each time point.

### p53 Mutation Analysis

Genomic DNA was extracted from tumor tissue using Quick-gDNA MiniPrep Kit (Zymo Research, Irvine, CA, USA). Sanger sequencing was performed by the Colorado Molecular Correlates Laboratory on p53 exons 4, 5, 6, 7, 8, and 10.

### Library Preparation and mRNA Sequencing (RNA-Seq)

Libraries were constructed using 1 µg total RNA following Illumina TruSeq RNA Sample Preparation Guide and the cDNA library was validated on the Agilent 2100 Bioanalyzer using DNA-1000 chip. Cluster generation was done on the Illumina cBot using a Single Read Flow Cell with a Single Read cBot reagent plate (TruSeq SR Cluster Kit). Sequencing of the clustered flow cell was performed on the Illumina HiSeq 2000 using TruSeq SBS v3 or v4 reagents. Single read sequencing of 100 or 125 base pairs were obtained from the sequencing machine. The raw data were analyzed in four steps: image analysis, base calling, sequence alignment, and variant analysis and counting. An additional step was required to convert the base call files (.bcl) into *_qseq.txt files. For multiplexed lanes/samples, a demultiplexing step is performed before the alignment step.

### RNA-Seq Analysis

Low-quality sequencing bases were trimmed before the alignment step. Trimmed sequencing reads were mapped against the human genome using the Tophat/cufflinks workflow. We used the UCSC reference annotation (hg19) as a guide, and allowing two mismatches for the initial alignment and two mismatches per segment with 25bp segments using Tophat (version 2.0.13). We employed Cufflinks (version 2.2.1) to assemble the transcripts using the RefSeq annotation as a guide and computed the transcripts’ FPKM values using the merged assembly as a guide. On average, 52.6 million (32.4–68.6 million) reads were sequenced per sample and 73.9% (66.6–85.8%) of the reads were aligned to the human genome. Gene set enrichment analysis (GSEA) was used to identify pathways enriched as previously described. We used the KEGG pathways as the gene set and performed 1,000 gene-set permutations to determine *p*-value. Pathways with *p* < 0.05 were considered significant from the analysis.

### TNBC Explant Subtyping

To predict the TNBC subtypes of the PDX models, we used the TNBCtype web tool (http://cbc.mc.vanderbilt.edu/tnbc) (20). RNA-seq data for these models were uploaded to the TNBCtype web tool. Based on the gene expression profiles, the TNBCtype was assigned to the individual sample as one of the six TNBC subtypes [basal-like BL1, basal-like BL2, immunomodulatory IM, mesenchymal M, mesenchymal stem-like (MSL), or luminal androgen receptor (LAR)] (20). If the gene expression of the sample contained signatures from more than one subtype, it was classified as UNS (unspecified). The corresponding correlation coefficient and permutated *p*-values of the TNBC subtyping were provided from the website.

### Immunoblotting Analysis

Protein was isolated from tumor samples. Forty micrograms of total protein was loaded onto a 4–20% gradient gel, electrophoresed, and then transferred to nitrocellulose using the i-Blot system (Invitrogen, Carlsbad, CA, USA). Membranes were blocked in blocking buffer and incubated overnight at 4°C with primary antibodies: 4E-BP1, p4E-BP1, and alpha-beta tubulin (Cell Signaling Technology, Beverly, MA, USA, catalog numbers 9644S, 9456S, 21485, respectively). Following primary antibody incubation, membranes were washed in TBS-Tween (0.1%) and incubated with secondary anti-rabbit or anti-mouse IgG1 horseradish peroxidase-linked antibody at 1:15,000 (Jackson ImmunoResearch, West Grove, PA, USA; catalog numbers 5151S and 5470S, respectively) for 1 h at RT. Blots were developed using the Odyssey Infrared Imaging System (LI-COR Biosciences, Lincoln, NE, USA). Experiments were performed in triplicate.

### Statistical Analysis

Treatment and vehicle groups *in vivo* were compared by unpaired parametric *t*-test with Welch’s corrections using a commercially available statistical program (Prism 4.0, Graph Pad).

## Results

### Antitumor Activity of ENMD-2076 in p53-Mutated TNBC PDX Models

**Figure 1:**
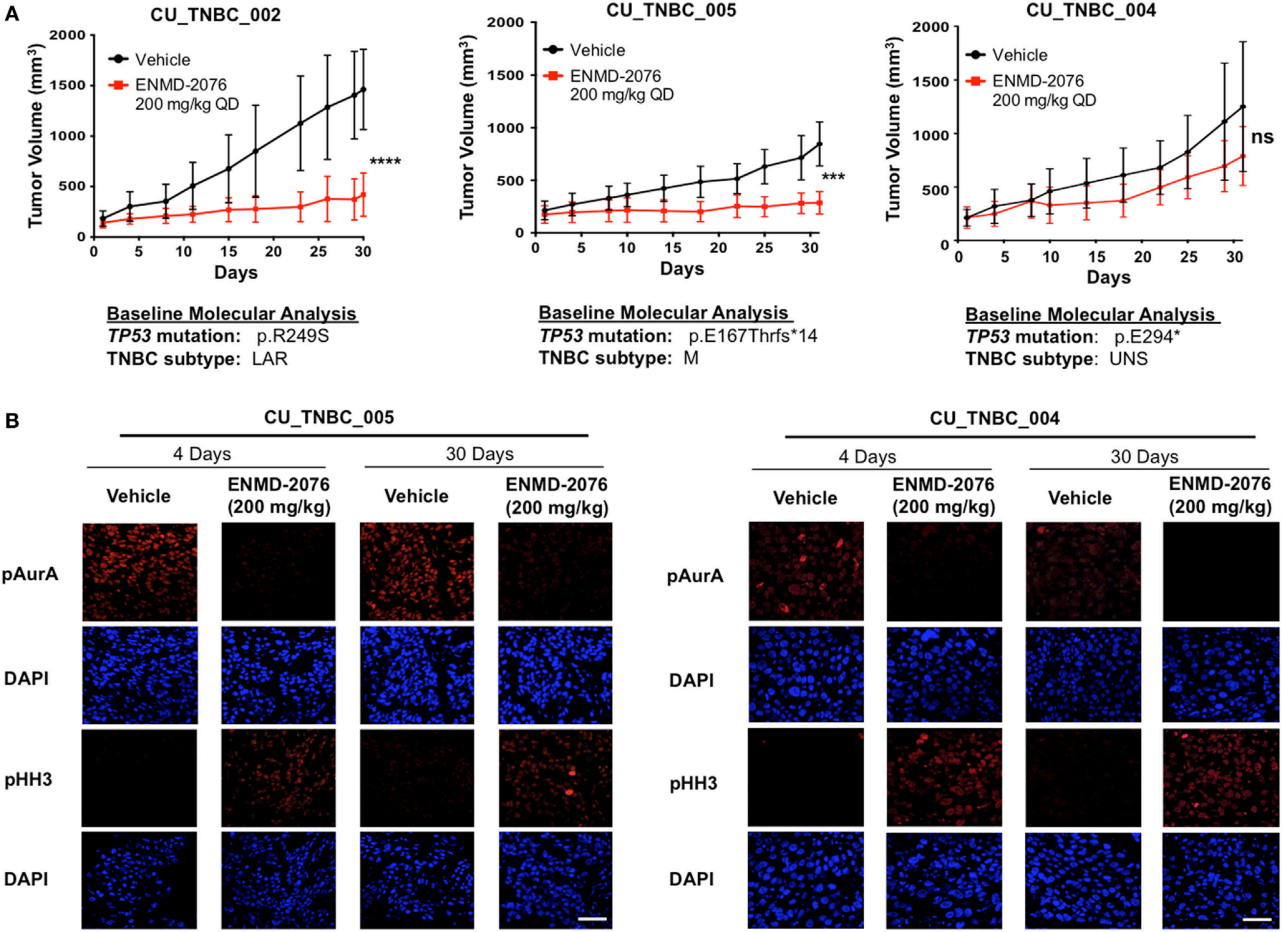
**Antitumor activity of ENMD-2076 in p53-mutated triple-negative breast cancer (TNBC) PDX models *in vivo***. **(A)** Effect of ENMD-2076 treatment (200 mg/kg once daily by oral gavage) in three PDX models. Tumor growth inhibition (TGI) *in vivo* was statistically significant in CU_TNBC_002 and CU_TNBC_005 (TGI 71.3%, ****p < 0.0001; TGI 66.1%, ***p < 0.001, respectively). Treatment with ENMD-2076 did not result in a statistically significant decrease in TGI in the CU_TNBC_004 model (TGI 37.0%, *p* > 0.05). The baseline molecular analysis is provided for each model, including p53 mutation and TNBC subtype using TNBCtype (20). **(B)** Immunofluorescence analysis of phospho-AurA (pAurA), phospho-histone H3, and DAPI control in CU_TNBC_005 and CU_004 tumors harvested from mice treated with ENMD-2076 or vehicle at the time points noted. Representative images were acquired using confocal microscopy at 60× magnification. Scale = 5 µm.

The antitumor activity of ENMD-2076 was evaluated *in vivo* against three TNBC PDX models. As depicted in Figure 1A, ENMD-2076 treatment resulted in statistically significant tumor growth inhibition in PDX models CU_TNBC_002 and CU_TNBC_005 at day 30 (*p* < 0.0001, TGI: 71.3%; and *p* < 0.001, TGI: 66.1%, respectively). The PDX model CU_TNBC_004 was intrinsically resistant to treatment with ENMD-2076 (TGI: 37.0%).

To further characterize the biology of these PDX models beyond the triple-negative phenotype at baseline, we determined the p53 mutational status and the predicted TNBC subtype using gene expression data and TNBCtype (20, 21). Sanger sequencing of TP53 exons 4–8 and 10 was performed on tumor tissue obtained prior to treatment and revealed mutations in p53 in all models. The mutations included: p.R249S (CU_TNBC_002), p.E167Tfs*14 in (CU_TNBC_005), and p.E294* (CU_TNBC_004). Mutations in p53 have been categorized as functional, partially functional, and non-functional based on transactivation capacity in diploid yeast models (22). The R249S TP53 mutation is a commonly occurring missense mutation resulting in loss of function in p53 transcription capacity (22). The mutations in p53 occurring in CU_TNBC_005 and CU_TNBC_004 are frameshift and early termination mutations, respectively, and presumably also result in impaired p53 transactivation capacity. Using TNBCtype, CU_TNBC_002 and CU_TNBC_005 were predicted to represent the LAR and mesenchymal (M) subtype, respectively. CU_TNBC_004 gene expression analysis using TNBCtype was inconclusive and predicted an unspecified group (UNS, unstable) (Figure 1A).

From these RNA-Seq data, we performed variant detection and identified exonic mutations present in CU_TNBC_002, CU_TNBC_005 and CU_TNBC_004 pretreatment, after treatment, and at the time of acquired resistance (if applicable). In this study, we focused on mutations that were non-synonymous and predicted to be deleterious by PolyPhen2 and SIFT algorithms, supported by >10 mutation reads. In the two models treated to acquired resistance (CU_TNBC_002 and CU_TNBC_005), we did not identify any new mutations at the time of acquired resistance. Similarly, we performed fusion detections using TopHat-Fusion on these acquired resistance models and did not identify any new fusions at the time of acquired resistance.

### Pharmacodynamic Effects of ENMD-2076 Treatment on pAurA and pHH3 Expression *In Vivo*

Treatment with ENMD-2076 200 mg/kg daily with continuous dosing resulted in a decrease in pAurA and an increase in pHH3 (Figure 1B). Changes were observed in sensitive (CU_TNBC_005) and resistant (CU_TNBC_004) models at day 4 and day 30. The observed increase in pHH3 is consistent with predominant inhibition of AurA as compared to Aurora B, leading to the inability of cells to complete successful mitosis (23–25).

### ENMD-2076 Treatment to Acquired Resistance in Sensitive PDX Models

**Figure 2:**
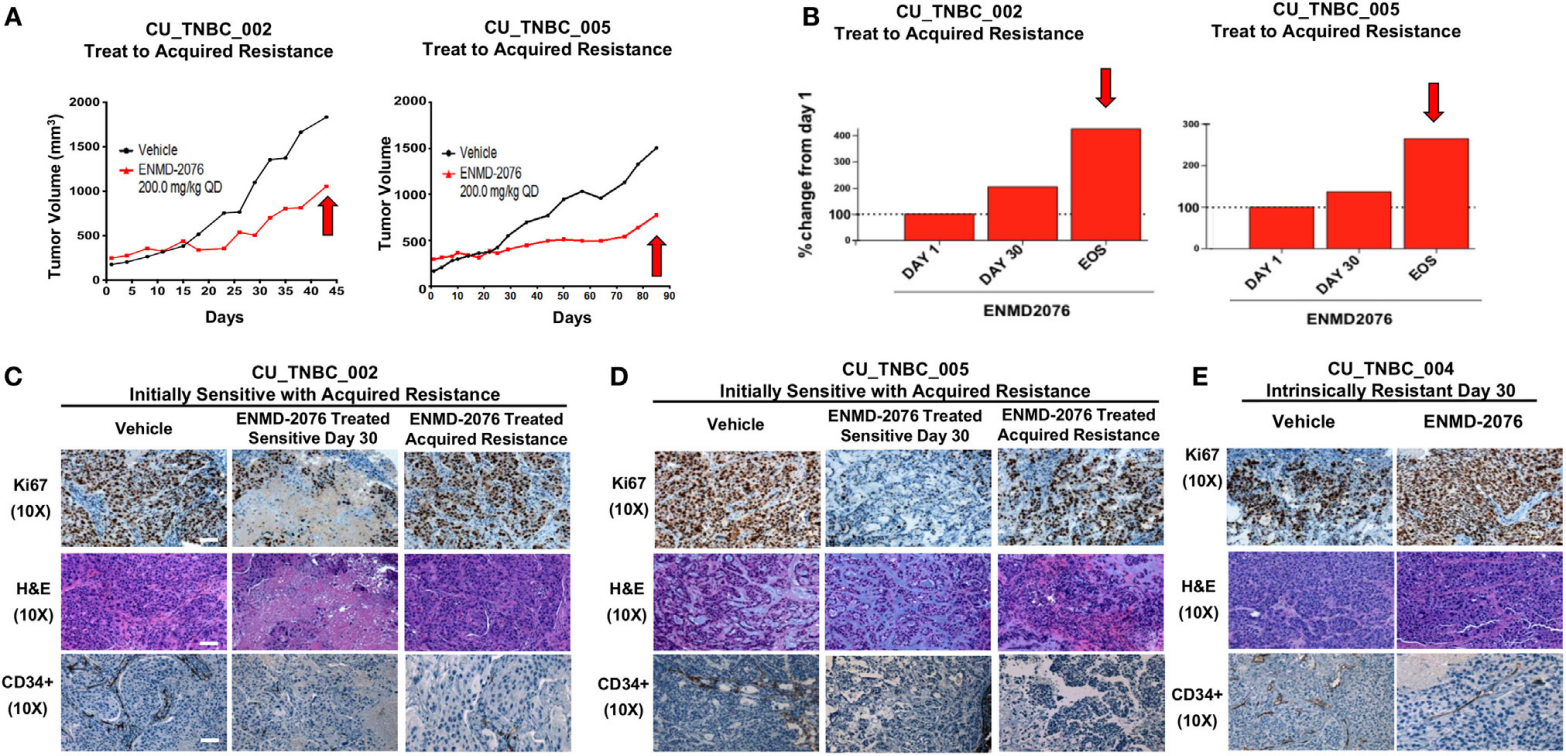
**Sensitive PDX models treated to acquired resistance to ENMD-2076 treatment**. **(A)** Tumor growth curves for PDX models CU_TNBC_002 and CU_TNBC_005 depicting individual ENMD-2076 treated tumors and vehicle controls selected for immunohistochemistry (IHC), IF, and genomic analysis. Acquired resistance was defined as a doubling in tumor volume from day 30 while receiving ENMD-2076 treatment. **(B)** % change from baseline for the selected tumors selected for acquired resistance in panel **(A)**. Arrows indicate acquired resistance tumors. **(C)** CU_TNBC_002 and **(D)** CU_TNBC_005 tumor samples obtained from vehicle, ENMD-2076 treated sensitive at day 30, and ENMD-2076 treated acquired resistance stained for hematoxylin/eosin (H&E), Ki67, and CD34. **(E)** CU_TNBC_004 tumor samples obtained from vehicle and ENMD-2076 treated at day 30. Representative images were taken using a Zeiss microscope at 10× magnification. Scale = 10 µm. EOS, end of study.

Treatment with ENMD-2076 was continued in the initially sensitive CU_TNBC_002 and CU_TNBC_005 models until the development of acquired resistance (Figures 2A,B). Figure 2A demonstrates the tumor growth curves for individual tumors selected for correlative studies, whereas the percent change from baseline for selected tumors is depicted in Figure 2B. We defined tumors with acquired resistance as exhibiting at least a doubling in tumor volume from day 30, when a statistically significant TGI was observed (*p* < 0.05). Individual tumors were selected for correlative studies based on overall mean tumor volumes to minimize necrotic tissue.

Treatment with ENMD-2076 resulted in a decrease in Ki67, a marker of cellular proliferation, in both sensitive models at day 30 as compared to vehicle control tumors (Figures 2C,D). At the time of acquired resistance to treatment, Ki67 increased to expression levels comparable to vehicle control. A decrease in Ki67 was not observed following ENMD-2076 treatment in the intrinsically resistant CU_TNBC_004 model (Figure 2E). ENMD-2076 treatment resulted in a decrease in microvessel density as measured by CD34 expression in all models and at all time points, indicating blockade of angiogenesis (Figures 2C–E).

### ENMD-2076 Treatment Results in an Induction of Apoptosis in Sensitive PDX Models *In Vivo*

**Figure 3:**
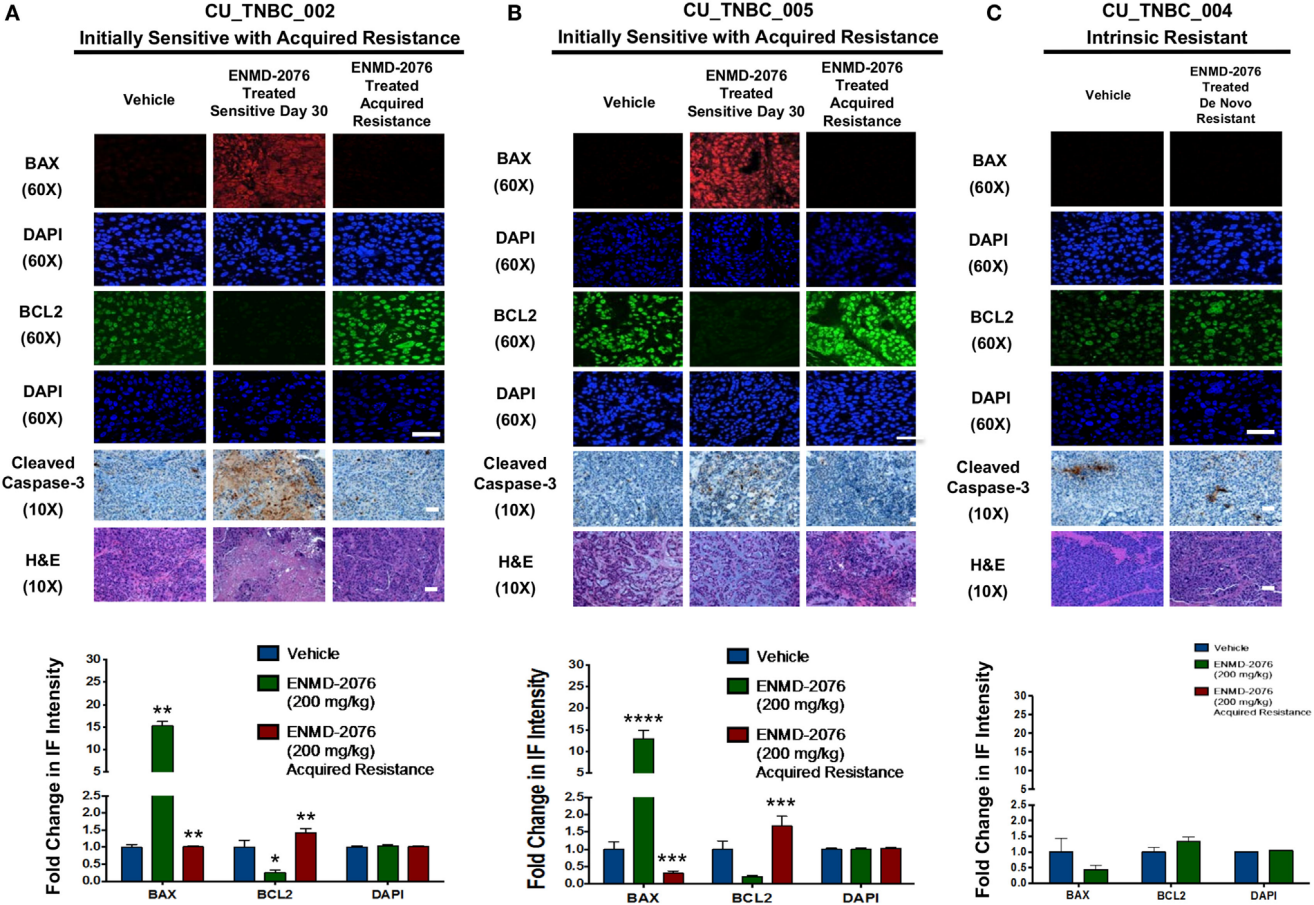
**Effects of ENMD-2076 treatment on markers of apoptosis in sensitive and resistance PDX models**. **(A,B)** Immunofluorescence (IF) analysis of tumors harvested from CU_TNBC_002 and CU_TNBC_005 treated with vehicle control, ENMD-2076 treated at day 30 (sensitive), and ENMD-2076 treated at the time of acquired resistance. Acquired resistance was defined as a doubling in tumor volume from day 30 while receiving ENMD-2076 treatment. ENMD-2076 treatment was administered at 200 mg/kg once daily by oral gavage. **(C)** IF analysis of tumors harvested from CU_TNBC_004 treated with vehicle control and ENMD-2076 (intrinsically resistant to ENMD-2076). Representative IF images were acquired using confocal microscopy at 60× magnification. Scale = 5μm. Representative IHC images were taken using a Zeiss microscope at 10× magnification. Scale = 10 µm. Quantification of mean pixel intensity of the IF analysis is shown. Sensitive tumor pixel intensity was compared to vehicle control and acquired resistance pixel intensity was compared to the sensitive tumor. *p < 0.05, **p < 0.01.

In order to understand the mechanism of tumor growth inhibition and decreased cellular proliferation observed with ENMD-2076 treatment *in vivo*, we investigated known mediators of apoptosis. As shown in Figures 3A,B, ENMD-2076 treatment in the CU_TNBC_002 and CU_TNBC_005 sensitive models led to a statistically significant increase in BAX (*p* < 0.01), a known mediator of intrinsic apoptosis, and a statistically significant decrease in the pro-survival marker BCL2 (*p* < 0.05) as compared to vehicle control. We also observed an increase in cleaved caspase-3 as measured by IHC, confirming apoptosis. At the time of acquired resistance to ENMD-2076 treatment in this model, we observed an increase in BCL2 (*p* < 0.01) and a decrease in BAX (*p* < 0.01) and cleaved caspase-3 compared to the sensitive tumors, consistent with loss of the previously observed apoptotic response. An increase in apoptosis was not observed in the intrinsically resistant model CU_TNBC_004 (Figure 3C).

### ENMD-2076 Treatment Results in Upregulation of p53 and p73 in Models Sensitive to the Antiproliferative and Proapoptotic Effects of ENMD-2076 *In Vivo*

**Figure 4:**
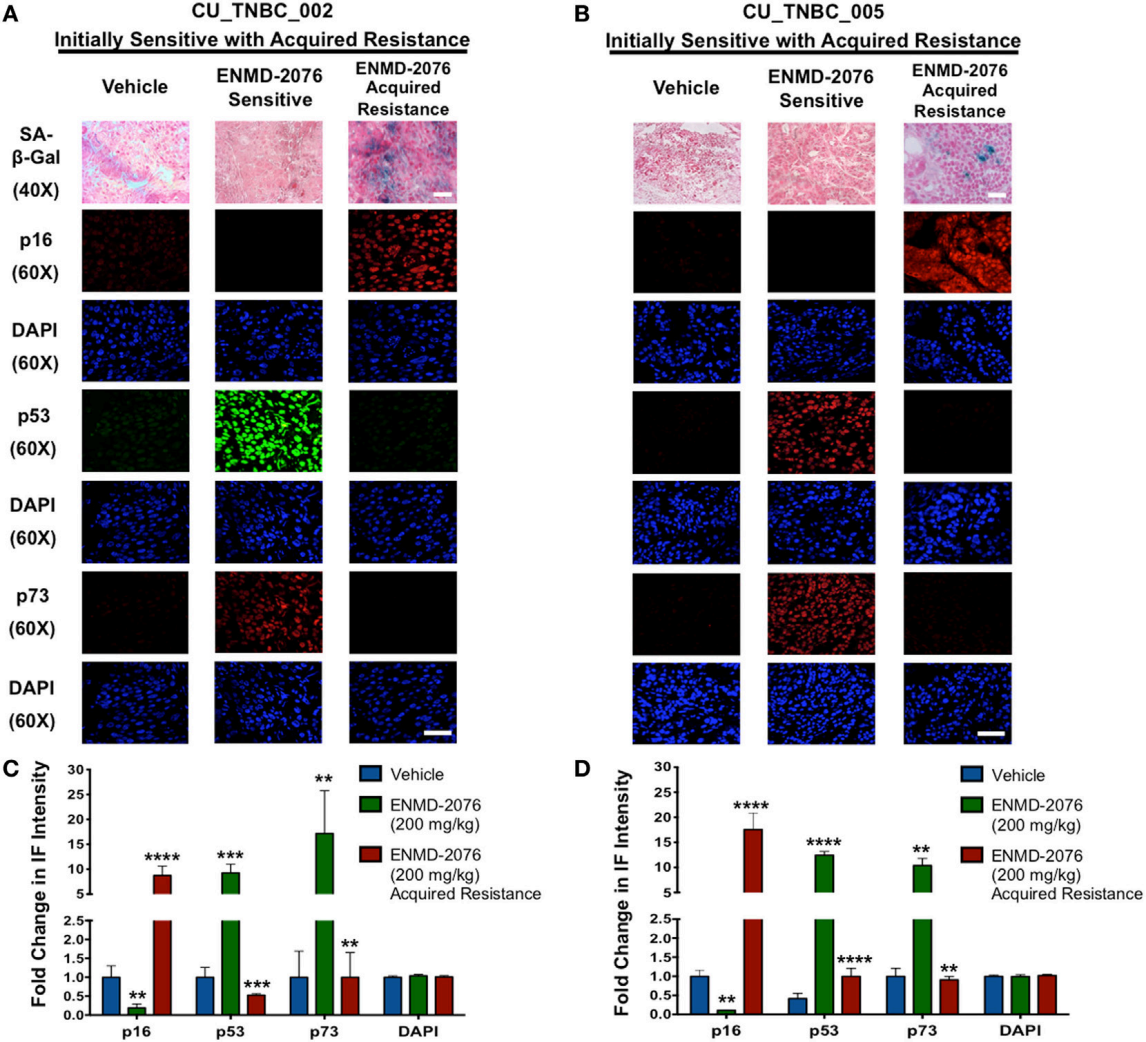
**Effects of ENMD-2076 treatment on SA-β-Gal, p16, p53, and p73 expression in PDX models exhibiting initial sensitivity to ENMD-2076 treatment and at the time of acquired resistance**. **(A,B)** Senescence and IF analysis of tumors harvested from CU_TNBC_002 and CU_TNBC_005 treated with vehicle control, ENMD-2076 treated at day 30 (sensitive), and ENMD-2076 treated at the time of acquired resistance. Acquired resistance was defined as a doubling in tumor volume from day 30 while receiving ENMD-2076 treatment. ENMD-2076 treatment was administered at 200 mg/kg once daily by oral gavage. Representative images for SA-β-gal expression were acquired using a Zeiss microscope at 40× magnification. Scale = 5μm. Note the presence of blue SA-β-gal staining in both models at the time of acquired resistance. Representative immunofluorescence (IF) images were acquired using confocal microscopy at 60× magnification. Scale = 5μm. **(C,D)** Quantification of mean pixel intensity of the IF analysis. Sensitive tumor pixel intensity was compared to vehicle control and acquired resistance pixel intensity was compared to the sensitive tumor. **p < 0.01, ***p < 0.001, ****p < 0.0001.

**Figure 5:**
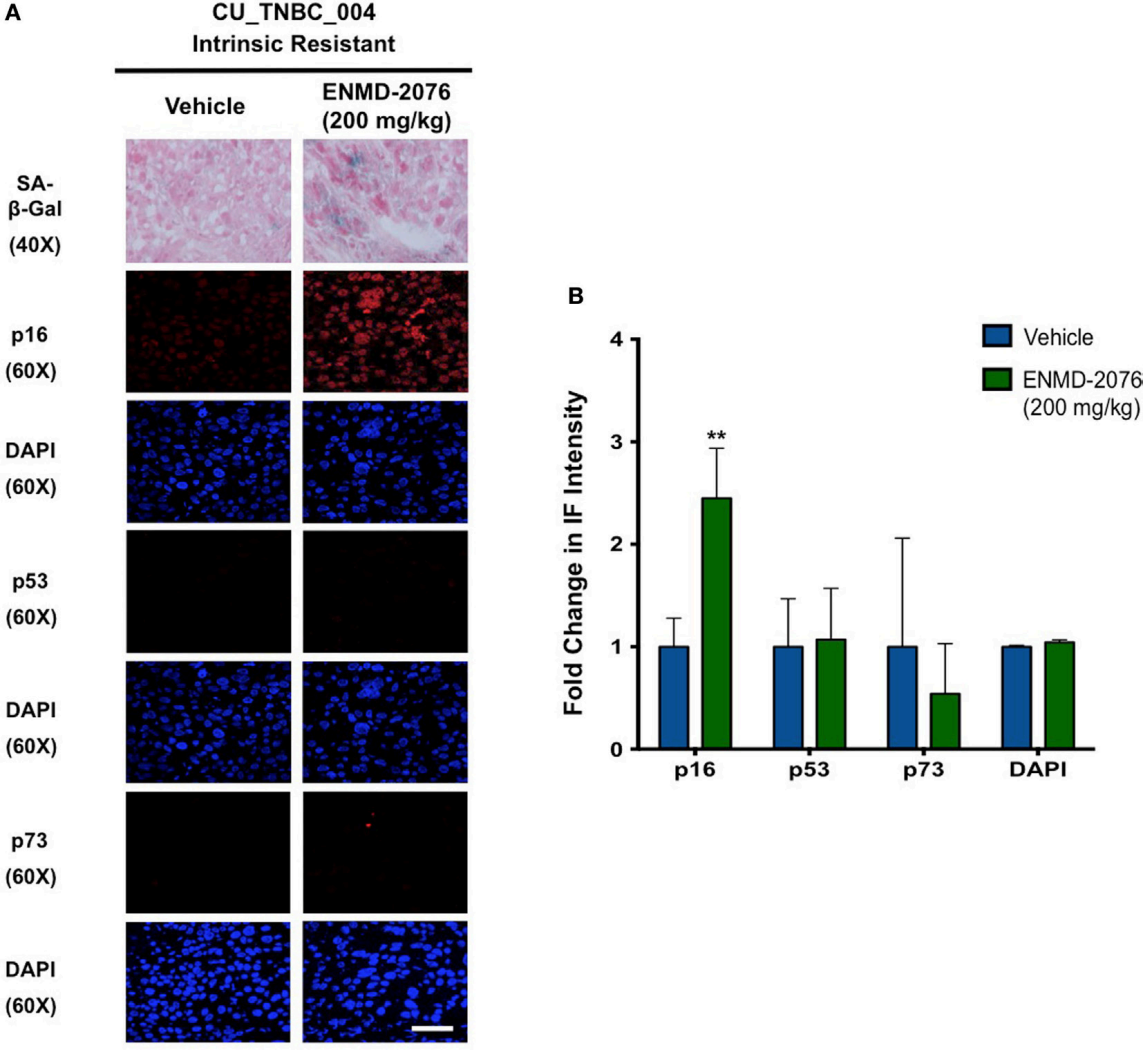
**Effects of ENMD-2076 treatment on SA-β-Gal, p16, p53, and p73 expression in PDX models exhibiting initial resistance to ENMD-2076 treatment**. **(A)** Senescence and immunofluorescence (IF) analysis of tumors harvested from CU_TNBC_004 treated with vehicle control and ENMD-2076 treated. ENMD-2076 treatment was administered at 200 mg/kg once daily by oral gavage. Representative images for SA-β-gal expression were acquired using a Zeiss microscope at 40× magnification. Scale = 5μm. Note the presence of blue SA-β-gal staining in the ENMD-2076 treated group. Representative IF images were acquired using confocal microscopy at 60× magnification. Scale = 5μm. **(B)** Quantification of mean pixel intensity of the IF analysis. **p < 0.01, ***p < 0.001, ****p < 0.0001.

Given the integral role of p53 and p73 in mediating cell cycle arrest and apoptosis in response to cellular stress, we evaluated expression of these proteins following ENMD-2076 treatment. As shown in Figures 4A,B, we observed a significant increase in p53 and p73 at day 30 of treatment in both sensitive models (CU_TNBC_002 and CU_TNBC_005). Quantification of pixel density was performed and confirmed a statistically significant increase for both models (Figures 4C,D). At the time of acquired resistance, expression of p53 and p73 was lost in both models. An increase in p53/p73 was not observed in response to ENMD-2076 treatment in the intrinsically resistant CU_TNBC_004 model (Figure 5).

### Intrinsic and Acquired Resistance to ENMD-2076 Treatment in PDX Models Is Associated with an Increase in Markers of Senescence

We have previously demonstrated that ENMD-2076 treatment leads to senescence in resistant breast cancer cell lines, particularly in those with decreased p53 expression (14). To confirm these observations in a clinically relevant model, we evaluated the functional senescence biomarkers, p16, and SA-β-gal, as well as phenotypic hallmarks of senescence (larger flattened cells, increased vacuoles, and larger nuclei) in the PDX models treated with ENMD-2076 (26–28). As depicted in Figures 4A,B, an increase in SA-β-gal and p16 staining was observed at the time of acquired resistance to treatment in the initially sensitive CU_TNBC_002 and CU_TNBC_005 models and following treatment in the resistant CU_TNBC_004 model (Figure 5).

### Changes in TNBC Subtype at the Time of Acquired Resistance to ENMD-2076 Treatment

**Figure 6:**
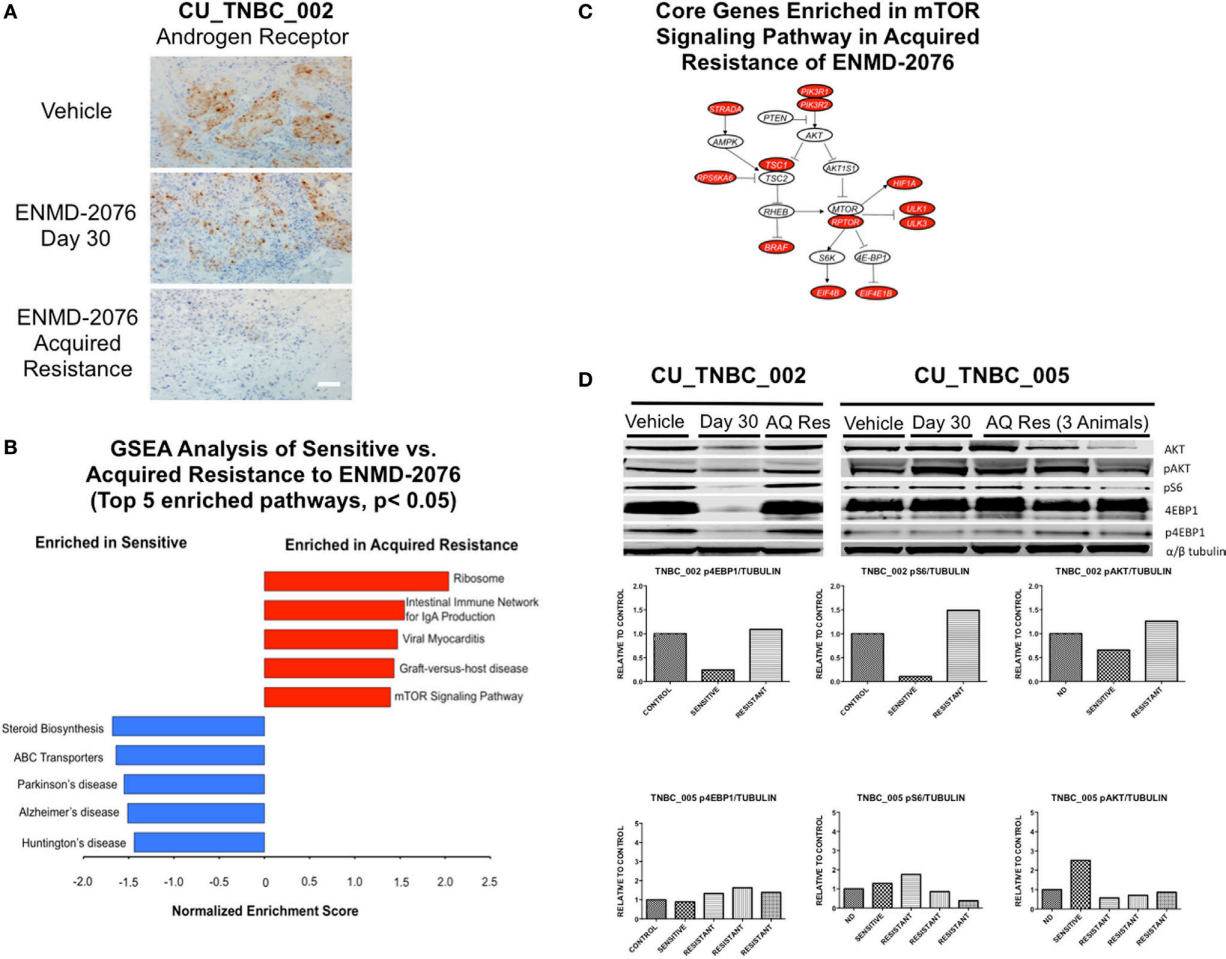
**Mechanisms of acquired resistance to ENMD-2076 treatment**. **(A)** IHC analysis of androgen receptor expression in tumors harvested from CU_TNBC_002 treated with vehicle control, ENMD-2076 treated at day 30 (sensitive), and ENMD-2076 treated at the time of acquired resistance. Samples were obtained from three animals at each time point. Representative images are shown. Acquired resistance was defined as a doubling in tumor volume from day 30 while receiving ENMD-2076 treatment. ENMD-2076 treatment was administered at 200 mg/kg once daily by oral gavage. Representative images were acquired using a Zeiss microscope at 10× magnification. Scale = 5μm. **(B)** Gene set enrichment analysis (GSEA) of the two acquired resistance vs. day 30 (sensitive) gene expression. Top five KEGG pathways with *p* < 0.05 were plotted. **(C)** The mTOR signaling pathway is a potential druggable pathway identified from GSEA analysis in the acquired resistance, and the core genes (colored in red) were illustrated. The pathway is adapted from KEGG mTOR signaling pathway (HSA04150). **(D)** Immunoblots from performed using tumor tissue obtained from vehicle, day 30, and acquired resistance confirming increased p4E-BP1 and pS6 at the time of acquired resistance in CU_TNBC_002. Quantification of signals from immunoblots was performed using Image Studio software, with identical areas measured for each band.

**Figure 7:**
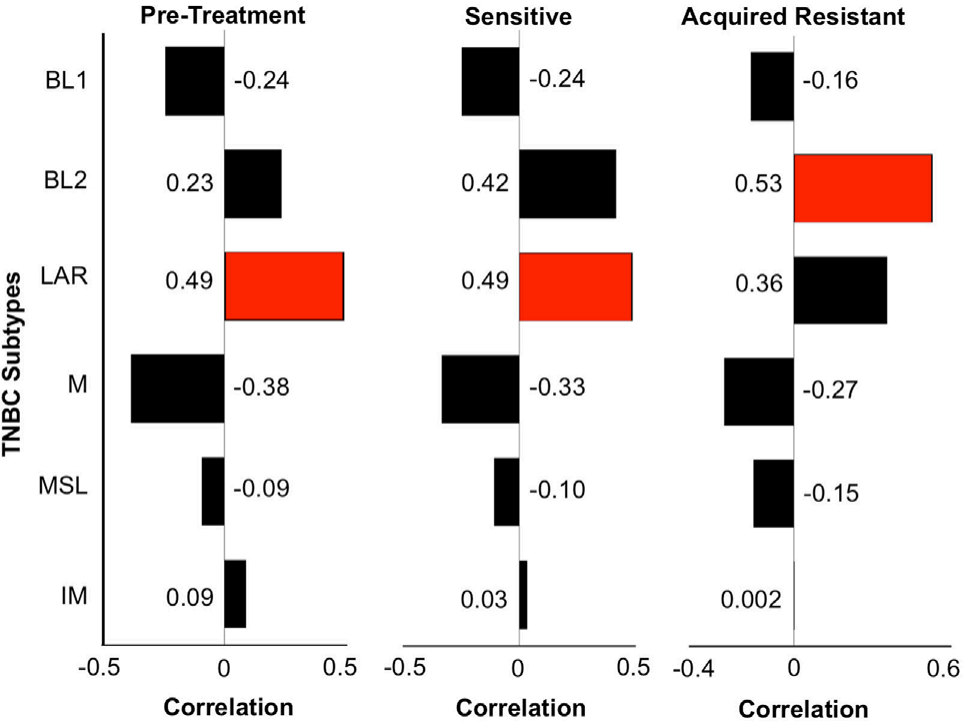
**Triple-negative breast cancer (TNBC) subtyping for CU_TNBC_002**. We performed TNBC subtyping on the CU_TNBC_002 model and computed the correlation of this model at the pretreatment, sensitive on treatment and acquired resistance time points against the six TNBC subtypes. At the pretreatment and sensitive time points, we observed luminal androgen receptor (LAR) as the dominant subtype followed by BL2. However, at the acquired resistance time point, BL2 was the dominant subtype followed by LAR. This subtype switching suggests expansion of BL2 subtype at acquired resistance in this model.

Genetic cluster analysis has been used to identify six TNBC subtypes: basal-like 1 and 2 (BL1 and BL2), immunomodulatory (IM), mesenchymal (M), MSL, and LAR subtypes (21). We used the TNBCtype online prediction tool and gene expression profiling data to predict the dominant TNBC subtype on samples obtained from CU_TNBC_002, CU_TNBC_005, and CU_TNBC_004 pretreatment, on treatment day 30, and at the time of acquired resistance in the two sensitive models (20) (Table 1). Interestingly, in the CU_TNBC_002 PDX model, we observed a subtype switch from LAR at baseline and day 30 to BL2 at the time of acquired resistance (*p* < 0.001). We performed IHC for the AR, confirming increased AR expression at baseline and day 30 as compared to the time of acquired resistance (Figure 6A). This subtype switch is likely a result of the clonal expansion of a BL2 cell population present in the tumor at baseline (Figure 7).

**Table 1:** **Triple-negative breast cancer (TNBC) molecular subtypes predicted using TNBCtype**.

TNBC PDX model	Timepoint	Predicted TNBC subtype	Correlation	*p-*Value
CU_TNBC_002	Pretreatment	LAR	0.471	<0.001
	On treatment: ENMD-2076 day 30	LAR	0.468	<0.001
	On treatment: ENMD-2076	BL2	0.433	<0.001
	Acquired resistance			
CU_TNBC_005	Pretreatment	M	NA	NA
	On treatment: ENMD-2076 day 30	UNS	0.339	<0.001
	On treatment: ENMD-2076	M	0.404	<0.001
	Acquired resistance			
CU_TNBC_004	Pretreatment	UNS	NA	NA
	On treatment: ENMD-2076 day 30	IM	0.435	<0.001

*LAR, luminal androgen receptor; BL2, basal-like 2; M, mesenchymal; UNS, unstable; IM, immunomodulatory*.

### GSEA and Pathway Analysis in Sensitive Models and Those with Acquired Resistance to ENMD-2076 Treatment

Gene set enrichment analysis was performed using RNA-Seq data generated from the PDX models treated with ENMD-2076 as a discovery exercise to identify pathways associated with intrinsic or acquired resistance to treatment (Figure 6B). We found the Ribosome and mTOR signaling pathways to be enriched at the time of acquired resistance and the steroid biosynthesis pathway to be enriched in sensitive samples. Core genes enriched in the mTOR signaling pathway in the acquired resistance models include PIK3R1, RPTOR, and RPS6KA6 (Figure 6C). Based on these data, we performed western blotting for key proteins in the mTOR pathway and observed variable changes in p4E-BP1, pAkt, and pS6 protein expression at the time of acquired resistance compared to sensitive-treated tumors (Figure 6D). These findings were not consistent across all models and time points and the investigation of the role of the mTOR pathway in resistance to ENMD-2076 should be further studies.

## Discussion

Despite extensive efforts to characterize the genomic heterogeneity of TNBC and identify novel therapeutic targets, TNBC remains a clinically aggressive breast cancer subtype with limited treatment options. The purpose of this study was to evaluate the antitumor activity of ENMD-2076, a multi-target AurA, and angiogenic kinase inhibitor, in p53-mutated TNBC PDX models and to identify molecular mechanisms of differentiated response to therapy. The study was designed to characterize patterns of terminal cell fate, namely apoptosis and senescence, in tumors sensitive to ENMD-2076 and those with intrinsic or acquired resistance. Additionally, we identified changes in key mediators of response to therapy, including p53, p73, BAX, and BCL2, and TNBC subtypes based on gene expression profiling.

ENMD-2076 has antitumor activity in p53-mutated TNBC PDX models, including models with p53 mutations predicted to result in impaired p53 function. While significant tumor regression was not observed at the doses tested in this study, ENMD-2076 treatment did result in durable statistically significant decreased tumor growth as compared to vehicle control. We believe that this is a relevant measure of antitumor activity in TNBC and can be equated to prolonged stable disease in patients, which is a clinically meaningful outcome given the aggressive nature of this disease.

We observed an increase in p53 and p73 protein expression following treatment with ENMD-2076 in sensitive models. This induction of p53 and p73 in response to ENMD-2076 treatment was not observed in tumors demonstrating intrinsic or acquired resistance. These data support a role for p53 and p73 in mediating sensitivity to ENMD-2076, likely in response to AKI-induced cellular stress and leading to the activation of apoptotic pathways. These findings are consistent with our previously published *in vitro* data demonstrating that shRNA knockdown of either p53 or p73 resulted in resistance to Aurora A inhibitors in TNBC (15).

While p53 is commonly mutated in TNBC, p73 has structural and functional similarity to p53, but is rarely mutated in cancer (29). Both p53 and p73 play critical roles in mediating cell cycle arrest and apoptosis in response to cellular stress stimuli, and p73 provides redundant functioning in the setting of mutated p53 (30, 31). The interaction of Aur A with p53 and p73 has also been characterized *in vitro*. In p53-deficient cell lines, Aur A overexpression leads to a decrease in p73 and downstream target gene expression, including PUMA, NOXA, and p21 (30). Furthermore, knockdown of endogenous Aurora A in this system by siRNA or AKI treatment results in an increase in p73 and transcriptional target gene expression (30). This is consistent with our findings demonstrating an increase in p73 in response to ENMD-2076 treatment in sensitive models harboring p53 mutations.

In TNBC, PDX tumors demonstrating intrinsic or acquired resistance to ENMD-2076 treatment, we observed the upregulation of markers of senescence, including SA-β-gal and p16, which is in contrast to the apoptotic response observed in sensitive models. Senescence has been classically described as a permanent cell cycle arrest that can function as tumor suppressive (27, 32). However, it has also been reported that senescent cells may secrete growth factors as part of the senescence-associated secretory phenotype, promoting tumorigenesis (33). Our findings *in vivo* of senescence associated with resistance to ENMD-2076 are consistent with our previous published data demonstrating an association between AKI treatment-induced senescence *in vitro* using the selective Aur A inhibitor MLN8237 or ENMD-2076 (14, 15). The mechanism of this differentiated response to treatment remains to be fully characterized and should be investigated in future studies.

Triple-negative breast cancer is currently defined clinically by the lack of expression of markers associated with response to targeted therapies in breast cancer, namely the estrogen receptor, progesterone receptor, and HER2 (3). This definition has resulted in a biologically heterogeneous disease made up of a number of subtypes based on the dominant gene expression pattern (21). However, even within the TNBC subtypes defined by gene expression profiling, there often exists further heterogeneity demonstrated by the reported dominant subtype and the runner-up subtype (20). In our study, we observed a switch in the dominant subtype in one model in response to treatment with ENMD-2076 and the development of acquired resistance. The subtype switch was from LAR to BL2 and accompanied by a decrease in AR expression by IHC at the time of acquired resistance. To our knowledge, this is the first description of such a subtype switch in TNBC in response to an anticancer treatment, however, is not surprising given what is know in other disease types regarding selective pressure induced by targeted therapies. We did not observe a subtype switch in CU_TNBC_005 at the time of acquired resistance; the M subtype was dominant at baseline and at acquired resistance. These findings support the conclusion that there are likely many different mechanisms of acquired resistance to ENMD-2076 in TNBC and individual mechanisms will differ based on genomic characteristics of the tumor at baseline. We plan to further investigate loss of AR expression as a potential mechanism of acquired resistance in AR positive TNBC through the development of additional AR positive TNBC PDX models and testing for AR in tumor tissue samples obtained from patients treated with ENMD-2076 in an ongoing Phase II clinical trial in metastatic previously treated TNBC (NCT01639248).

In this study, we also observed the enrichment of core genes in the mTOR signaling pathway at the time of acquired resistance. This was accompanied by variable changes in p4EBP1, pS6, and pAKT *in vivo* in different models. Others have shown that the proapoptotic activity of AKIs is dependent on an inactivated status of the mTOR pathway (34, 35). Furthermore, in colorectal cancer cell lines isolated from tumors resistant to the AKI MK-8745, an increase in phosphorylation of mTOR and Akt was observed and these cells underwent apoptosis in response to mTOR and Akt inhibition (35). The association of mTOR pathway activation and resistance to ENMD-2076 should be further investigated using patient samples from ongoing clinical trials. The combination of the Aur A inhibitor alisertib and the mTOR inhibitor MLN0128 is also being investigated in an ongoing Phase I clinical trial with a planned expansion cohort in metastatic TNBC designed to include serial tumor biopsies and functional imaging to further characterize the biologic response to combination therapy (NCT02719691).

## Ethics Statement

This study was performed in accordance with recommendations from the NIH guidelines for the care and use of laboratory animals in a facility accredited by the American Association for Accreditation of Laboratory Animal Care with approval by the University of Colorado Institutional Animal Care and Use Committee prior to initiation of experiments.

## Author Contributions

AI, JK, AC, TP, KR, and RH made substantial contributions to the acquisition and analysis of data, drafting and revising the work for important intellectual content, final approval of the version to be published, and agreement to be accountable for all aspects of the work in ensuring that questions related to the accuracy or integrity of any part of the work are appropriately investigated and resolved. JD, PK, CS, AT, JT, and SE made substantial contributions to the overall design of the work, analysis and interpretation of data, drafting and revising the work for important intellectual content, final approval of the version to be published and agreement to be accountable for all aspects of the work in ensuring that questions related to the accuracy or integrity of any part of the work are appropriately investigated and resolved.

## Conflict of Interest Statement

Scientific consulting CASI pharmaceuticals (SE); research funding from CASI pharmaceuticals (JD). The other authors declare no conflict of interest.
